# Shared Environmental Crises as an Impetus for Improving India-Pakistan Relations

**DOI:** 10.7759/cureus.76736

**Published:** 2025-01-01

**Authors:** Bilal Irfan, Maryam Aftab, Aneela Yaqoob

**Affiliations:** 1 Center for Bioethics, Harvard Medical School, Boston, USA; 2 Center for Surgery and Public Health, Brigham and Women's Hospital, Boston, USA; 3 Department of Neurology, University of Michigan Medical School, Ann Arbor, USA; 4 Department of Internal Medicine, Dow University of Health Sciences, Karachi, PAK; 5 Infectious Diseases, Beaumont Hospital, Taylor, USA

**Keywords:** air quality, health politics, india, pakistan, public health

## Abstract

The recent rise in air pollution in India and Pakistan offers an avenue for public health experts in the two countries to work together to mitigate risks. The effects of climate change transcend borders, and limited resources in a volatile environment open a door for stakeholder involvement. Amid the deteriorating pulmonary health, school closures, and compromised livelihoods, there is an argument to be made that joint healthcare initiatives, harmonized policy frameworks, and integrated environmental monitoring can serve as catalysts for easing longstanding geopolitical tensions. Such collaborative endeavors may include shared research on immediate and long-term health impacts, the joint development of clean technologies, and the alignment of industrial and vehicular emission standards. There are actionable frameworks that leverage shared health priorities and environmental stewardship that can be leveraged as an impetus for change and can be co-led by Pakistani and Indian equivalents of a health ministry, medical universities, and teaching hospitals. Such synergistic approaches not only improve public health outcomes but also foster stability, trust, and enhanced collaboration in a region profoundly affected by climatic and political uncertainties. However, challenges like political resistance, funding constraints, and resource limitations may hinder progress, highlighting the importance of clear stakeholder roles and multilateral support.

## Editorial

The recent surge in air pollution across northern India and Pakistan has reached unprecedented levels, posing severe health risks and necessitating immediate, coordinated action. On November 18, 2024, New Delhi’s Air Quality Index (AQI) soared to 1,758, significantly exceeding the “hazardous” threshold of 500 [1]. Likewise, Lahore recorded AQI levels surpassing 1,900 on November 7, 2024, underscoring a critical environmental crisis [1]. In some parts of the Punjab region, the recorded air quality was more than 50 times higher than the World Health Organization’s recommended safe limit [2]. These extremely high AQI values often indicate elevated concentrations of particulate matter (PM2.5 and PM10), which have been linked to acute and chronic respiratory conditions, cardiovascular diseases, and certain types of cancer. For perspective, on November 19, 2024, the AQI in New York was 38, while it was 28 in London, and 22 in Paris [1].

Key contributors to this pollution surge include vehicular emissions, industrial discharges, and extensive agricultural residue burning [3]. Winter weather exacerbates these issues, as cooler temperatures and stagnant air masses trap pollutants near ground level, causing a dense, health-endangering smog that engulfs urban centers. Considering the combined population of the Pakistani province of Punjab and the Islamabad Capital Territory along with the Indian states of Haryana, Himachal Pradesh, Punjab, and Delhi and the Union Territory of Chandigarh, an estimated 220 million people are affected - likely an underestimation given the region’s population density. In many urban areas of this cross-border region, population density can exceed 1,000 people per square kilometer, placing enormous strain on healthcare systems and amplifying the socioeconomic burden when environmental disasters strike.

School and workplace closures are one immediate consequence of these pollution levels. These closures disrupt education, diminish productivity, and can exacerbate existing inequalities when families lose income or children lose valuable learning time. Long-term health implications include increased risks of pulmonary diseases, respiratory ailments, allergies, and irritation of the eyes and throat [4]. In addition, higher exposure to fine particulate matter can contribute to chronic obstructive pulmonary disease (COPD), heart disease, stroke, and certain cancers over time. Healthcare facilities, public health officials, and medical personnel share a collective responsibility during such environmental catastrophes, which transcend political boundaries. The longstanding territorial conflict between India and Pakistan has persisted for decades, with the heavily militarized border clearly visible from space, yet this pressing environmental threat demands a departure from historical tensions [5].

The severity of the current crisis underlines the urgency for collaborative action. Delaying such efforts could intensify the health burden, overstretch healthcare infrastructures, and incur higher economic costs due to lost labor and mounting medical expenses. Global comparisons to lower AQI readings in cities like New York and London serve as an important reminder that effective governance, robust policy implementation, and shared technological solutions may be necessary to curb pollution. Such coordinated regional action is not just an aspirational endeavor, but a necessity for the health of the region. Effective bilateral collaboration may focus on strengthening healthcare partnerships, advancing environmental monitoring, and implementing joint policy measures. Historically, India and Pakistan have collaborated on environmental and water-related data sharing. For example, hydrological data exchange helped provide flood warnings in advance, thereby potentially averting larger calamities. Such precedents provide a roadmap for reinitiating and expanding cooperation, especially in health emergencies that do not respect borders. Both countries face common climate-related challenges, including infectious diseases and non-communicable conditions aggravated by environmental degradation. Prompt, coordinated responses can improve public health outcomes and mitigate the impact of climate change while simultaneously fostering trust and easing geopolitical friction (Table 1). 

**Table 1:** Proposed framework for India-Pakistan collaboration on air pollution SAARC: South Asian Association for Regional Cooperation; UNEP: United Nations Environment Programme; WHO: World Health Organization

Focus area	Actionable solutions	Potential outcomes
Air quality monitoring	Establish a cross-border air quality monitoring network and data-sharing	Improved response to hazardous conditions
Bilateral task force	Form a joint task force to harmonize air pollution standards, curb shared sources, and promote sustainable alternatives to stubble burning. However, obstacles such as political resistance and resource limitations must be addressed, potentially through multilateral funding or incremental pilot programs	Enhanced air quality through unified pollution regulations, reduced emissions, and adoption of sustainable agricultural practices
Healthcare and research collaboration	Cross-border training for healthcare practitioners, supported by shared resources, ventilators, and air purifiers. This can be sustained through government allocations, philanthropic support, or cost-sharing mechanisms. Organize collaborative studies on immediate and long-term health effects, such as respiratory and cancer risks, and compare data with other less-polluted regions	Improved medical response capacity and healthcare access for effective crisis management. Better scientific evidence for policymaking, stronger advocacy for environmental standards, and the establishment of a shared repository of health data
Clean technology sharing	Establish technology-sharing agreements for cleaner industrial processes and renewable projects, including co-development of solar and wind farms. Such arrangements could draw inspiration from inter-country agreements (e.g., transboundary haze initiatives in other regions)	Sustainable energy solutions, economic benefits, reduced agricultural emissions and pollution
Public awareness and media involvement	Launch joint campaigns, in partnership with mass or social media entities, to educate farmers on eco-friendly practices and raise awareness about pollution’s health impacts and prevention. Leverage schools, community centers, and online platforms for dissemination	Enhanced public understanding, adoption of protective behaviors, and increased community involvement
Policy alignment	Negotiate agreements to align environmental policies, such as vehicle emission standards and industrial waste regulations	Streamlined policies curbing cross-border pollution sources
Localized cooperation	Foster city-level collaborations (e.g., Delhi-Lahore, Amritsar-Sialkot) for pollution control and support community-driven initiatives like plantation drives	Strengthened ties between local governments and communities, leading to increased grassroots engagement. Local cultural and political differences may affect how policies are implemented, which requires outreach programs tailored to individual communities
Facilitation	Involve organizations like the United Nations or SAARC for dialogue on shared interests and collaborate with UNEP and WHO for expertise	Reduced political tensions, enhanced cooperation on less contentious issues, and increased transparency and accountability

Shared healthcare services and coordination between hospitals, academic institutions, and the health ministries of India and Pakistan can be a catalyst for reducing tensions. It would not be the first instance of collaboration between India and Pakistan on environmental issues. For decades, until 2019, the two states shared hydrological data, providing critical water flow and flood information. This information exchange was pivotal in enabling timely flood warnings and mitigation efforts, demonstrating tangible outcomes of cooperative engagement. There is no shortage of health crises shared by both countries, where collaborations and coordination in responses can promote better outcomes for the people of India, Pakistan, and the disputed territory of Kashmir, a better objective rather than exacerbating disputes on the allocation of crucial water and land resources such as the Indus River. It is imperative that such initiatives are fostered, nurtured, and enhanced at the government and local level with a degree of urgency because the climate will not wait for them. Effective funding models could include international grants, joint government allocations, or partnerships with non-governmental organizations, ensuring that resource constraints do not derail long-term collaboration. Looking ahead, delays in implementing these bilateral measures could worsen environmental hazards and inflate healthcare costs, while foreclosing opportunities to de-escalate regional tensions through shared problem-solving. By merging public health imperatives with environmental management, both states have an opportunity to address some of the leading causes of morbidity (such as respiratory illnesses, cardiovascular conditions, and infectious diseases) in a multifaceted way. Collaborative action, which ranges from cross-border task forces and research programs to coordinated policy alignments, holds a pathway to not just a cleaner, but a lasting vision for a shared future in a historically fraught region. Regional cooperation on air pollution embodies an opportunity to improve public health, advance economic well-being as a consequence, and potentially soften geopolitical hostilities. While there are numerous obstacles, including but not limited to financing, political reluctance, and differing policy frameworks, the demonstrated success of past India-Pakistan collaborations offers a foundation upon which to build. It is something that healthcare advocates, physicians, and public health experts in both countries can work towards promoting and bridging understanding. Stakeholders, be they national and local governments, multilateral agencies, public health experts, industries, research institutions, and the media, all have roles to play in championing initiatives for the health of our shared world. The climate crisis, escalating every year, demands immediate and decisive steps. By acting promptly, India and Pakistan can transform a shared environmental threat into a platform for constructive engagement, thereby charting a more hopeful future for their citizens.
